# Combined dorsal plus ventral double buccal mucosa graft in bulbar urethral reconstruction

**Published:** 2008

**Authors:** Arun Chawla, Sreedhar Reddy, Joseph Thomas

**Affiliations:** Department of Urology, Kasturba Medical College, Manipal, India. E-mail: urologyarun@yahoo.com

## SUMMARY

This is a retrospective study of the use and efficacy of combined dorsal and ventral double buccal mucosal graft (BMG) in bulbar urethral reconstruction. A total of 48 males, of mean age 35 years, underwent this procedure from March 2002 to June 2006. The stenotic urethral segment was opened ventrally and dorsal urethra was incised in midline to create an elliptical area over the tunica albuginea. Fibrotic tissue was partially excised from urethral margins taking care to preserve the urethral plate. Dorsal inlay BMG (av. length 2.36 cm) was placed and quilted. Subsequently ventral onlay BMG (av. length 4.68 cm) was sutured to the urethral lateral margins and spongioplasty was performed. The average stricture length was 3.65 (2-10) cm and mean follow-up was 22 (13-59) months. The technique was successful (normal voiding without any need of postoperative procedures) in 43 (89.6%) patients. Of the five (10.4%) failures recurrences developed within 12 months after surgery and were mainly at proximal or distal anastomotic sites. VIU was successful in four while one patient needed perineal urethrostomy. The authors concluded that combined double patch of buccal mucosa was much better in patients having tight strictures with very narrow and scarred urethral plate. This technique provides wider neourethra than a single patch graft substitution.

## COMMENTS

Anastomotic urethroplasty is indicated only for short bulbar urethral strictures (<2 cm) and carries the risk of urethral chordee, impaired sensation of glans, and stricture recurrences.[1] For selected cases of long strictures, graft augmented anastomotic procedures were done which involved the resection of worst fibrotic area and placement of the graft.[2] But as in any anastomotic urethroplasty, this technique too has limitations in very long segment strictures. Currently, buccal mucosa graft substitution urethroplasty is the most preferred option for long bulbar urethral strictures. Barbagli *et al.* has shown that placement of grafts on the dorsal, ventral, or lateral surface of urethra provided the same success rate and stricture recurrence was uniformly distributed in all the patients.[3] In tight strictures, the placement of a buccal graft is a difficult exercise. McAninch *et al.* has proposed placement of 20-25 cm wide graft in near or completely obliterated urethra.[4] However, increase in the width of the graft is associated with enhanced risk of diverticulum formation.[4] The authors in their study have demonstrated that combined placement of double dorsal and ventral buccal mucosa graft creates a neourethra which is sufficiently wide, stable and maintains axial continuity of the urethral lumen. Their technique is founded on the principle of preservation of the integrity of the urethral place and its vascularity.[5] The technique does allow the partial removal of fibrotic urethral tissues without compromising the adequacy of the urethral lumen, thus adding further to the success.

The technique is the first to be described for the repair of tight bulbar strictures. This offers significant advantage over graft augmented urethroplasty and full circumferential urethral reconstruction by one stage graft tube in the management of tight and long strictures. However, the study is retrospective, has limitation of having a short follow-up and lacks comparison with other standard simple buccal graft substitution to document its possible advantages.
